# A sticky solution

**DOI:** 10.7554/eLife.00655

**Published:** 2013-04-02

**Authors:** David Gresham

**Affiliations:** 1 **David Gresham** is at the Center for Genomics and Systems Biology, Department of Biology, New York University, New York, United Statesdgresham@nyu.edu

**Keywords:** Multicellularity, Experimental evolution, Evolution of cooperation, S. cerevisiae

## Abstract

Selection favours single-celled mutants that stick together when a sugar needed for growth is in short supply, suggesting that multicellular life may have evolved as a by-product of selection for more efficient usage of resources.

**Related research article** Koschwanez JH, Foster KR, Murray AW. 2013. Improved use of a public good selects for the evolution of undifferentiated multicellularity. *eLife* **2**:e00367. doi: 10.7554/eLife.00367

**Image** Individual yeast cells (yellow) evolve to form multicellular clumps (magenta and green)

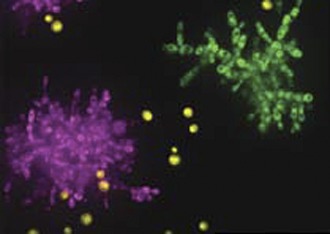

We typically think of single-celled microbes as being solitary creatures endowed with an innate self-sufficiency. But at some point during their evolutionary history, single-celled microbes made a transition to multicellularity that ended their autonomy and initiated a steady march towards increasing complexity. This evolutionary leap of faith occurred independently several times, and understanding what caused single-celled microbes to relinquish a life of solitude is a longstanding problem in evolutionary biology. Now, writing in *eLife*, John Koschwanez and Andrew Murray at Harvard University, and Kevin Foster at the University of Oxford, demonstrate that multicellularity can be selectively advantageous for microbes when sticking together solves a problem that cannot be solved by single-celled individuals (Koschwanez et al., 2013).

In this study the motivation for the budding yeast, *Saccharomyces cerevisiae*, to stick together is table sugar. *S. cerevisiae* metabolizes the common sugar, sucrose, by first converting it to glucose and fructose *outside* the cell in a process called hydrolysis. This reaction is performed by a protein called invertase, which is secreted by the yeast cells, and the glucose and fructose are then imported into the cells, where they are used to meet the metabolic requirements for growth. Because the sucrose is broken down in the extracellular environment, the cell that supplies the invertase is not necessarily the same cell that will import the reaction products. This doesn't matter when there are enough cells and enough sucrose—all cells will produce invertase and all cells can use the glucose and fructose produced by the collective activity of the population. However, in a low sucrose environment, it is likely that the fraction of these products that diffuse away before they can be imported is high enough to mean that the local sugar concentration available to each yeast cell is insufficient for growth.

An engineer would solve this problem in one of three ways: increase the amount of invertase that each cell makes so as to increase the amount of hydrolyzed sucrose; import the sucrose into the cell before hydrolysis; or increase the local density of cells in order to increase the local concentration of hydrolyzed sucrose. The current study and previous work by the same group (Koschwanez et al., 2011) show that these three rationally designed solutions all work. However, engineered solutions may not be accessible or beneficial during evolution, so the relevant question is: which of these routes will be favoured by adaptive evolution?

To address this question, Koschwanez, Foster and Murray performed parallel evolution experiments. Cells were introduced into a low sucrose environment and propagated for several weeks. At the end of this experiment, all cells were considerably better at growing in a low sucrose environment than they had been to start with, indicating that mutation and selection had worked efficiently to increase fitness. Moreover, nearly all of the cells now formed multicellular clumps, demonstrating that adaptive evolution had arrived at the same solution as rational engineering. The majority of cells had also increased their expression of the invertase gene, whereas none had acquired the ability to import sucrose. So, of the three solutions envisaged, adaptive evolution chose two.

**Figure 1.**
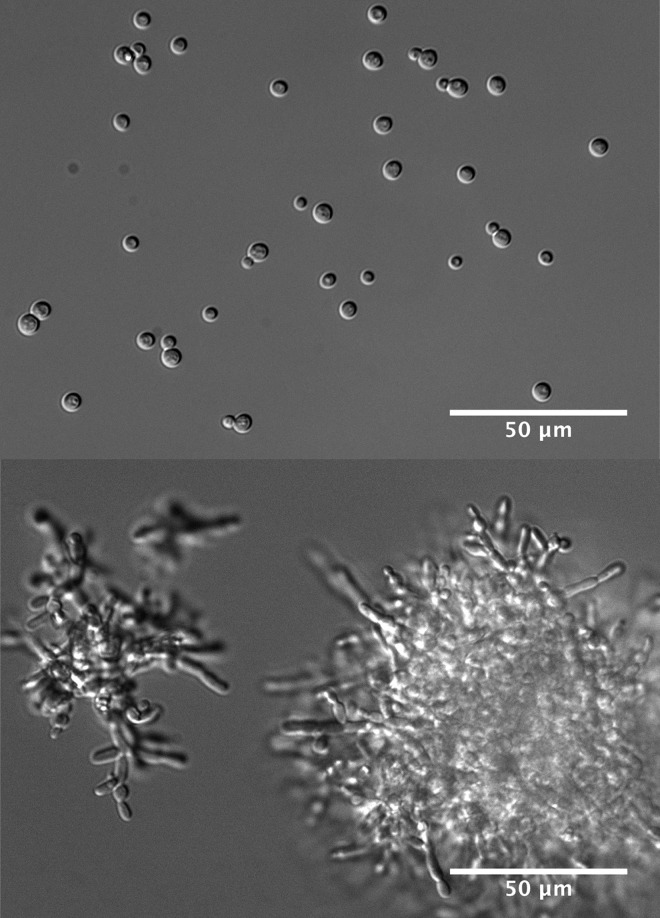
The budding yeast *S. cerevisiae* feeds on sucrose that it metabolizes outside the cell using the enzyme invertase, which it secretes into its surroundings. The breakdown products—glucose and fructose—are then imported into the cell and used to drive growth. If sucrose is in short supply and the density of single cells is low, the cells cannot capture enough of the glucose and fructose to initiate growth (upper panel). Under these conditions, mutations that cause the cells to form undifferentiated multicellular clumps (lower panel) confer an advantage, by increasing the local concentration of glucose and fructose available to cells. Selection for these mutations may lead ultimately to the evolution of multicellularity.

FIGURE CREDIT: IMAGES: [JOHN KOSCHWANEZ].

To identify the genetic basis of these acquired traits, Koschwanez et al. used the awesome power of yeast genetics and next generation sequencing. They identified more than 1500 mutations in the evolved cells, of which 80 seemed to help cells grow faster in low sucrose. The group of causal mutations included changes in a gene called *ACE2*, which encodes a protein required for completion of cell separation, and which results in a clumpy phenotype when defective (Gresham et al., 2006). Mutation of *ACE2*, plus microscopic analysis of cell clumps, reveals that the clumping phenotype arises from a defect in the separation of daughter cells after division, and not as a result of cells adhering to each other. While the underlying cause of increased invertase expression requires further investigation, Koschwanez et al. also identified mutations that were responsible for beneficial traits that they had not anticipated prior to the experiment. These included mutations that increased the expression of genes responsible for the transport of glucose and fructose into cells, and mutations that reduce the tendency of cells to cease growth when nutrient availability is low.

The results of this study present a route to undifferentiated multicellularity (that is, clumps of cells that all have similar properties) that contrasts with recent results reported by Nicole King and co-workers (Alegado et al., 2012). In that study, the unicellular choanoflagellate, *Salpingoeca rosetta*—one of the closest living relatives of animals—was shown to form multicellular colonies in the presence of a signaling molecule produced by bacteria. As in yeast, undifferentiated multicellularity occurs as a result of incomplete cell separation. However, in *S. rosetta* this appears to be a physiological response, rather than one that is acquired by mutation. Nevertheless, an intriguing possibility is that *S. rosetta* may have evolved to form multicellular colonies in the presence of bacteria in order to make themselves more efficient predators of those bacteria. If this were the case, the force driving undifferentiated multicellularity in both *S. rosetta* and yeast would be an increase in the efficiency of resource usage, which may be a common theme underlying the emergence of multicellularity.

Koschwanez et al. speculate that selection for clumping of unicellular organisms may have ultimately led to multicellularity. However, it is clear that selecting for mutants that stick together is relatively easy, and can be achieved in a single mutational step. Wild budding yeast are frequently clumpy and, in many of the commonly used laboratory strains, experimenters have selected for loss of clumpiness to enable easier manipulation of cells. Indeed, during long-term evolution experiments in yeast, selection for clumpy cells can occur intentionally (Ratcliff et al., 2012) and, sometimes, unintentionally (Lang et al., 2011). However, multicellular organisms are more than a conglomerate of undifferentiated cell types, and specialization of cell types is a defining feature of multicellular organisms. The challenge now is to design experiments that address the question of how multicellular organisms with distinct cell types might have emerged from single-celled organisms.

This process could have happened along two evolutionary trajectories. First, selection for undifferentiated multicellularity may have enabled the subsequent development of specialized cell types. Second, many microbes—including budding yeast (Gimeno et al., 1992) and *S. rosetta* (Dayel et al., 2011)—can exist as distinct morphological types with different characteristics. Perhaps existing in a multicellular form comprised of several physiologically distinct cell types could be beneficial in some environments. In any case, employing the powerful combination of rational engineering and long-term selection used by Koschwanez et al. is likely to prove fruitful in testing these different models.
